# Identification, Evolutionary and Expression Analysis of *PYL-PP2C-SnRK2s* Gene Families in Soybean

**DOI:** 10.3390/plants9101356

**Published:** 2020-10-14

**Authors:** Zhaohan Zhang, Shahid Ali, Tianxu Zhang, Wanpeng Wang, Linan Xie

**Affiliations:** 1 College of Life Sciences, Northeast Forestry University, Harbin 150040, China; zhangzhaohan@nefu.edu.cn (Z.Z.); shahidali@nefu.edu.cn (S.A.); tianxuzhang@nefu.edu.cn (T.Z.); wangwanpeng@nefu.edu.cn (W.W.); 2 Key Laboratory of Saline-Alkali Vegetative Ecology Restoration, Ministry of Education, College of Life Science, Northeast Forestry University, Harbin 150040, China

**Keywords:** soybean, PYL, PP2C, SnRK2, ABA signal pathway, differential expression, gene family

## Abstract

Abscisic acid (ABA) plays a crucial role in various aspects of plant growth and development, including fruit development and ripening, seed dormancy, and involvement in response to various environmental stresses. In almost all higher plants, ABA signal transduction requires three core components; namely, PYR/PYL/RCAR ABA receptors (PYLs), type 2C protein phosphatases (PP2Cs), and class III SNF-1-related protein kinase 2 (SnRK2s). The exploration of these three core components is not comprehensive in soybean. This study identified the *GmPYL-PP2C-SnRK2s* gene family members by using the JGI Phytozome and NCBI database. The gene family composition, conservation, gene structure, evolutionary relationship, *cis*-acting elements of promoter regions, and its coding protein domains were analyzed. In the entire genome of the soybean, there are 21 *PYLs*, 36 *PP2Cs*, and 21 *SnRK2s* genes; further, by phylogenetic and conservation analysis, 21 *PYLs* genes are classified into 3 groups, 36 *PP2Cs* genes are classified into seven groups, and 21 *SnRK2s* genes are classified into 3 groups. The conserved motifs and domain analysis showed that all the *GmPYLs* gene family members contain START-like domains, the *GmPP2Cs* gene family contains PP2Cc domains, and the *GmSnRK2s* gene family contains S_TK domains, respectively. Furthermore, based on the high-throughput transcriptome sequencing data, the results showed differences in the expression patterns of *GmPYL-PP2C-SnRK2s* gene families in different tissue parts of the same variety, and the same tissue part of different varieties. Our study provides a basis for further elucidation of the identification of *GmPYL-PP2C-SnRK2s* gene family members and analysis of their evolution and expression patterns, which helps to understand the molecular mechanism of soybean response to abiotic stress. In addition, this provides a conceptual basis for future studies of the soybean ABA core signal pathway.

## 1. Introduction

Soybean (*Glycine max* L. *Merr*) is an essential leguminous crop that provides the human diet with an essential protein source, livestock feed, and industry biodiesel [1]. However, the sustainability of soybean yields is challenged by expected changes in the climate with prolonged drought, salinity, and high temperature in many areas of the world [2]. Many innate survival mechanisms have evolved to respond to these adverse conditions, a crucial metabolic process called abscisic acid accumulation [3]. Abscisic acid (ABA) is a key regulator for plant stress-adaptation; the ABA signaling pathways are made up of many important turnover mechanisms in response to ever-changing environmental conditions [4]. The PYL-PP2C-SnRK2s family of proteins are the main members of the ABA core signaling pathway and play an important role in response to abiotic stress-mediated by ABA. PYL is located at the top of the ABA signal transduction pathway, mainly regulated by inhibiting type 2C protein phosphatase (PP2C) [5]. In the absence of ABA, PYL protein does not bind to PP2C, so PP2C activity is very high, thus preventing the activation of SNF1-Related protein kinases2 (SnRK2) and its downstream factors; in the presence of ABA, PYL binds to PP2C and inhibits its activity [6,7]. The inhibited PP2C release SnRK2, phosphorylated SnRK2 activating the ABA response element-binding factor (ABFs), and initiates a downstream response to stress [8,9]. ABA signal transduction pathway plays a key role in plant response to drought and salt stresses [10,11]. At present, the PYL family of the model plant *Arabidopsis thaliana* has been studied comprehensively. 14 PYL proteins with highly conserved amino acid sequences have been identified in *A. thaliana* [12]. Some PYL protein functions have been successfully verified, such as overexpression of *AtPYR1*, *AtPYL1*, *AtPYL2*, and *AtPYL3* genes that can improve drought resistance and water use efficiency of *A. thaliana* [13]. In *A. thaliana*, the PYR1 and PYL2 are the key receptors for stomatal closure induced by abscisic acid [14]. Overexpression of *AtPYL5* reduces the transpiration rate and reduce water loss, oxidative stress damage, and increase photosynthesis rate under drought stress [15]. Overexpression of *AtPYL4*, *AtPYL8*, and *AtPYL13* genes can enhance drought tolerance in *A. thaliana* [16,17,18,19]. Although the soybean *GmPYLs* gene family members are already identified [20], the bioinformatics analysis and expression characteristics of the soybean *GmPYLs* gene family have not been reported. The SnRK2 is the key component of ABA signal transduction pathways; each SnRK2 protein contains three functional domains, such as a conserved Ser/Thr kinase domain, adenosine triphosphate (ATP) binding domain [21], and a diverse regulatory C-terminal. The members of the SnRK2s family have been identified in various plant species, including *Triticum aestivum* (*TaSnRK2.3*, *2.4*, *2.7*, and *2.8*) [22,23], *Oryza sativa* (*SAPK1–10*) [24,25], *A. thaliana* (*AtSnRK2.1–2.10*), *G. max* (*GmSnRK2.1–2.22*) [26]. Different studies demonstrated ABA hypersensitivity after PP2C loss-of-function mutant, indicating its important role in ABA signaling pathway. PP2C proteins belong to monomer enzymes, and their functions depend on Mg^2+^ and Mn^2+^. The catalytic domain of PP2C proteins is located at either the C-terminus or N-terminus in eukaryotes [27]. Further study has shown that the catalytic domain regions of eukaryotic PP2C proteins are conserved, while the non-catalytic domain regions have diverse amino acid sequences [28]. However, to the best of our knowledge, bioinformatics analysis of *PYL-PP2C-SnRK2s* gene families in soybean is not comprehensive.

To further explore soybean genome information at the whole genome level, and elaborate the structure and function of *GmPYL-PP2C-SnRK2s* gene families, in the present study, the *GmPYL-PP2C-SnRK2* family in soybean, their phylogenetic relationship, chromosomal distribution, gene structure, protein motifs, and expression patterns in various tissues have helped to better understand the molecular mechanisms and the role of core components in the ABA signaling pathway. Furthermore, this study should help the breeder to manipulate and improve the yield and quality of soybean under different stress conditions.

## 2. Results

### 2.1. Identification and Nomenclature of GmPYL-PP2C-SnRK2s Gene Families

To identify the *GmPYL-PP2C-SnRK2s* gene family members in soybean genome using *A. thaliana* and *O. Sativa PYL-PP2C-SnRK2s* gene family as queries, we named the homologous soybean genes of each *GmPYL-PP2C-SnRK2s* by searching Blastp against NCBI, a total of 21 *GmPYLs*, 36 *GmPP2Cs*, and 21 *GmSnRK2* genes. The genes were named *GmPYL1~GmPYL21*, *GmPP2C1~GmPP2C36*, and *GmSnRK2.1~GmSnRK2.21*, respectively. Furthermore, the length, amino acid sequence of genes, and position of these genes on chromosomes were identified (Table S1). The coding sequence length of the 21 *GmPYLs* genes varies from 534 to 801 bp, but the encoded protein length ranges from 177 to 266 amino acids. The coding sequence length of 36 *GmPP2Cs* genes varies between 732 to 2163 bp, and the protein length ranges from 243 to 720 amino acids. The coding sequence length of 21 *GmSnRK2s* genes varies from 1011 to 1113 bp, and the length of the encoded protein is almost the same, ranging from 336 to 370 amino acids. The number of *PYL-PP2C-SnRK2s* did not vary substantially between *A. thaliana* (*PYL*:14, *PP2C*:12, *SnRK2*:10) and *O. sativa* (*PYL*:13, *PP2C*:12, *SnRK2*:10), but the amount of *PYL-PP2C-SnRK2* in *G. max* (*PYL*:21, *PP2C*:36, *SnRK2*:21) was significantly higher than that in *A. thaliana* and *O. sativa*.

### 2.2. Phylogenetic Analysis of GmPYL-PP2C-SnRK2s Gene Families

**Figure 1:**
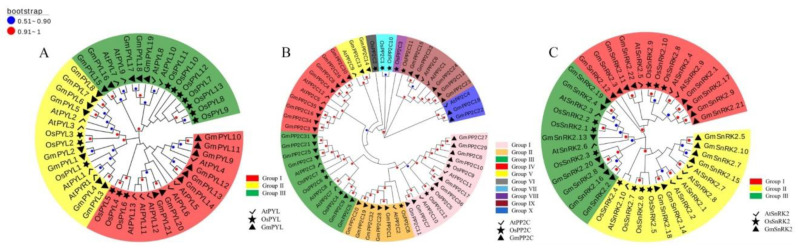
Phylogenetic trees of *PYLs* (**A**), *PP2Cs* (**B**), and *SnRK2s* (**C**) genes in *G. max*, *A. thaliana*, and *O. sativa* by using the complete protein sequences. The phylogenetic trees were constructed, the Maximum Likelihood was adopted using MEGA 10.0 software, with 1000 bootstrap replicates. In *GmPYLs* and *GmSnRK2s* genes, the group I–III, and in *GmPP2Cs* genes, I–X use different colors to distinguish them.

To classify and determine the evolutionary relationship of *PYL-PP2C-SnRK2s* genes, the amino acid sequences of PYLs, PP2Cs, and SnRK2s proteins from *G. max*, *A. thaliana*, and *O. sativa* were selected to construct three phylogenetic Maximum Likelihood trees. The name and ID of *PYL-PP2C-SnRK2s* genes in *O. sativa* and *A. thaliana* are shown in Table S2. According to the branching characteristics and bootstrap values of phylogenetic trees, a total of 48 *PYLs* from these three species were clustered into three subgroups (I–III), including 21 *GmPYLs* (*G. max*), 14 *AtPYLs* (*A. thaliana*), and 13 *OsPYLs* (*O. sativa*) (Figure 1A). A total of 60 *PP2Cs* from these three species were clustered into ten subgroups (I–X), including 36 *GmPP2Cs* (*G. max*), 12 *AtPP2Cs* (*A. thaliana*), and 12 *OsPP2Cs* (*O. sativa*) (Figure 1B), And a total of 41 *SnRK2s* from these three species were clustered into three subgroups (I–III), including 21 *GmSnRK2s* (*G. max*), 10 *AtSnRK2s* (*A. thaliana*) and 10 *OsSnRK2s* (*O. sativa*) (Figure 1C). Meanwhile, *GmPYL17*, *GmPYL18*, and *GmPYL19* were phylogenetically close to *AtPYL8* and *AtPYL10*, and *GmPP2C13* and *GmPP2C14* were phylogenetically close to *AtPP2C9*. Furthermore, *GmSnRK2.4* and *GmSnRK2.19* were phylogenetically close to *AtSnRK2.3*. There may be similarities in gene structure, protein structure, and functional domain among the same group of genes *PYLs*, *PP2Cs*, and *SnRK2s* in *A. thaliana* and *G. max* (Figure 1).

### 2.3. Evolutionary Conservation and Gene Structure of GmPYL-PP2C-SnRK2s

**Figure 2:**
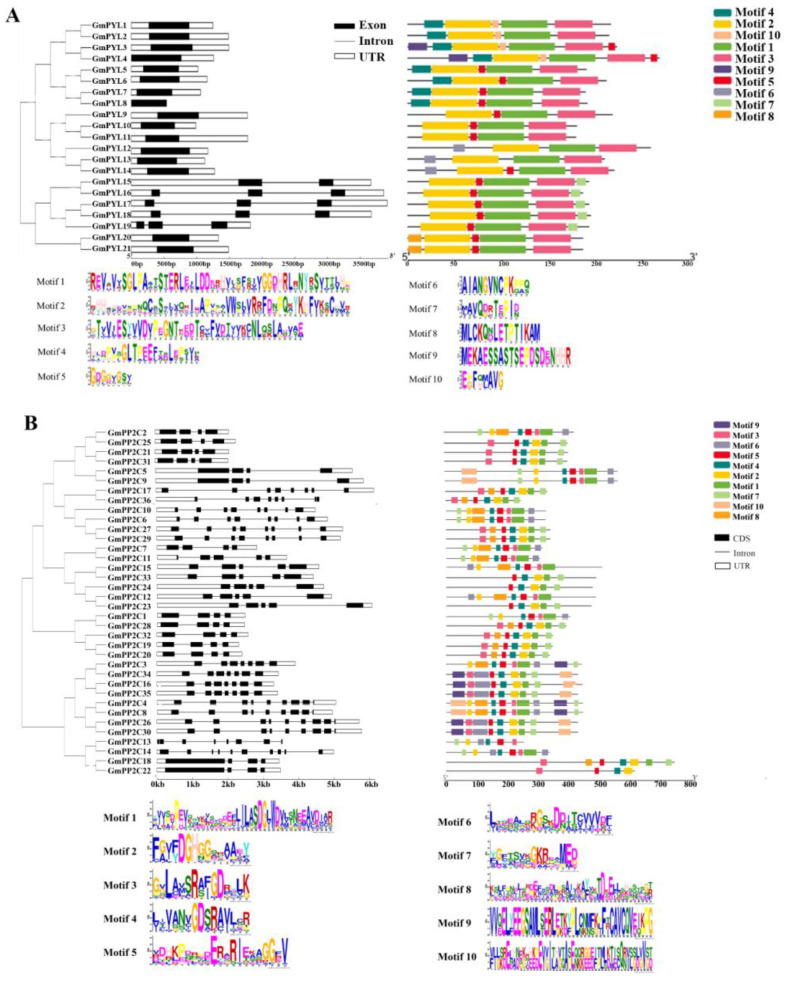

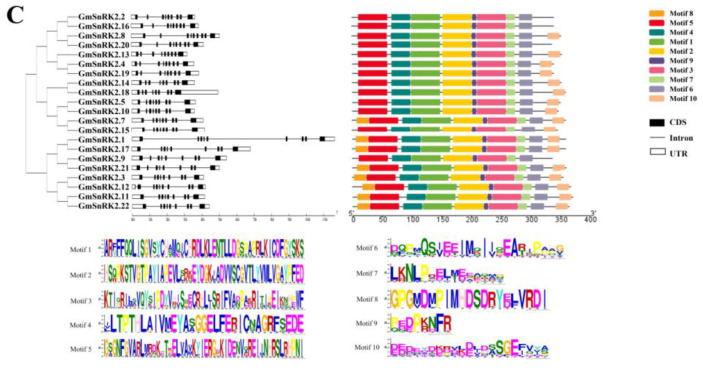
The gene structure and conserved motifs of *GmPYLs* (**A**), *GmPP2Cs* (**B**), and *GmSnRK2s* (**C**) based on phylogenetic relationships. All motifs were identified using the MEME website with the complete amino acid sequences of *GmPYLs*, *PP2Cs*, and *SnRK2s*. For example, in A, the phylogenetic tree (left) was constructed using the MEGA10.0 program with the complete amino acid sequences of the 21 GmPYLs proteins; Exon and intron structure analyses of *GmPYLs* genes were performed by the GENE Structure website(middle). The white boxes, black boxes, and the black lines indicate UTRs region, exons, and introns. The length of the amino acid, exon, and intron can be inferred by the ruler at the bottom—conserving motif distribution map of the *GmPYLs* gene performed by TBtools (right). The 10 predicted motifs are represented by different colored boxes, Motif LOGO showing below.

We used the GENE Structure display website (http://gsds.gao-lab.org/) to draw *GmPYL-PP2C-SnRK2s* gene structure online, and show 16 *GmPYLs* genes have no intron, except *GmPYL15*, *GmPYL16*, *GmPYL17*, *GmPYL18*, and *GmPYL19* (Figure 2A) [29]. In the *GmPP2Cs* family, 14 genes have 4 introns, while *GmPP2C14* has 10 introns, *GmPP2C13* lacks the UTR region. Almost all *GmSnRK2s* have 9 introns; only *GmSnRK2.12* has 10 introns.

For conservative motif analysis, used MEME website (http://meme-suite.org/) showed that 10 conserved motifs (motif1~motif10) were identified in GmPYL-PP2C-SnRK2s proteins, respectively [30]. The width, sites, and E-value of GmPYL-PP2C-SnRK2s proteins and their conserved motifs are shown in Table S3. The conserved motif’s width, approximately 8–50 amino acids, conserved motif LOGO, is shown in Figure 2. Genes in the same branch of the phylogenetic tree have a similar conservative motif. 

**Figure 3:**
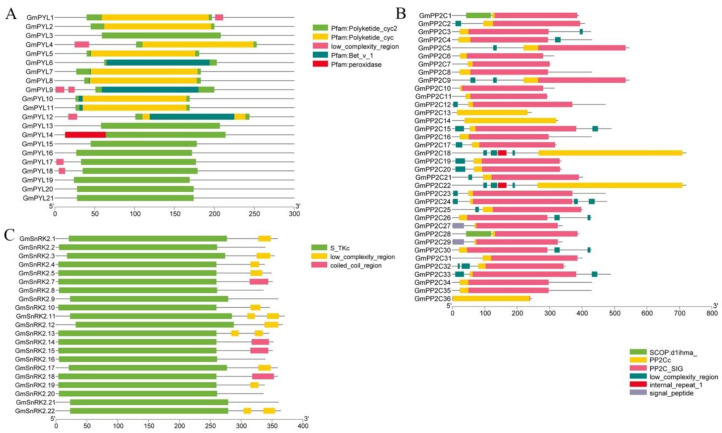
Analysis of the conserved domains *GmPYLs* (**A**). *GmPP2Cs* (**B**), and *GmSnRK2s* (**C**) by using the SMART website. All *GmPYLs* gene contains Pfam: Polyketide_cyc2 domain. All *GmPP2Cs* genes contain PP2Cc domain, and further, each *GmSnRK2s* contains a major serine-threonine (Ser/Thr) kinase catalytic domain (S_TKc).

Using “Batch SMART” plug-in in the TBtools software (Version 1.055) upload amino acid sequence, we found that all *GmPYLs* genes include the Polyketide_cyc2 domain, some *GmPYLs* genes contain Polyketide_cyc and Pfam: Bet_v_1 domain, only *GmPYL14* contains Pfam: peroxidase domain, which indicates that *GmPYL14* may have different functions in the *GmPYLs* gene family (Figure 3A). All 36 *GmPP2Cs* genes contain PP2Cc domains (Figure 3B). Each *GmSnRK2s* gene contains a major serine-threonine (Ser/Thr) kinase catalytic domain (S_TKC) (Figure 3C). The phosphatase domain of the PP2C-type consists of 10 beta-strand segments and 5 alpha-helix segments and comprises a pair of detached subdomains. The first is a small beta-sandwich with strand beta1 packed against strands beta2 and beta3; the second is a larger beta-sandwich in which a four-stranded beta-sheet pack against a three-stranded beta-sheet with flanking alpha-helices. And each *GmSnRK2* contains a major serine-threonine (Ser/Thr) kinase catalytic domain (S_TKc). The catalytic domain of serine/threonine protein kinases is predominantly alpha-helical and displays functional and structural similarity with the ATP-grasp fold and PIPK. Anti-parallel beta-sheets form the alpha and beta folds. It is a phosphotransferase that contains a conserved catalytic center. There is a glycine-rich area of residues near a lysine residue involved in ATP binding at the N-terminal end. There is an aspartic acid residue in the center of the STKc catalytic domain that drives the catalytic enzyme (Figure 3C), SCOP d1ihma domain is distinctive to *GmPP2C1*, *GmPP2C28*. Furthermore, the coiled-coil region is differential to *GmSnRK2.7*, *GmSnRK2.14*, and *GmSnRK2.15* nearly all genes contain a low complexity region.

### 2.4. Chromosomal Localization and Collinearity Analysis of GmPYL-PP2C-SnRK2s Gene Families

**Figure 4:**
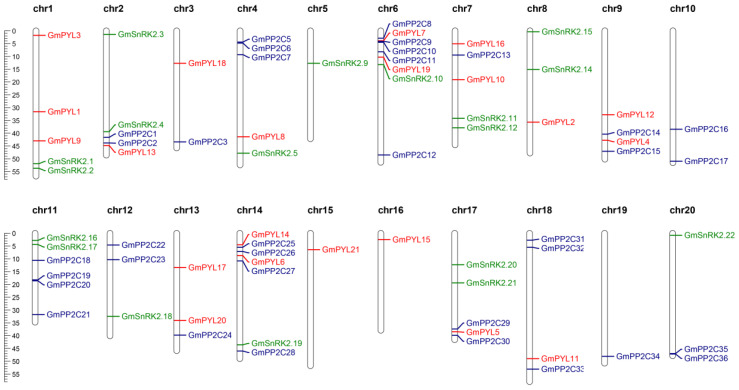
Chromosomal localization of *GmPYLs*, *GmPP2Cs*, and *GmSnRK2s* genes. 21 *GmPYLs* genes were distributed on chromosomes 1–9 and 11–18, the 36 *GmPP2Cs* genes are located on chromosomes 2–4, 6, 7, 9–14, and 17–20; further, the 21 *GmSnRK2s* genes are located on chromosomes 1, 2, 4–8, 11, 12, 14, 17 and 20, respectively. The chromosome numbers are indicated at the top of each vertical bar. The ruler indicates the length of the chromosome/Mb.

**Figure 5:**
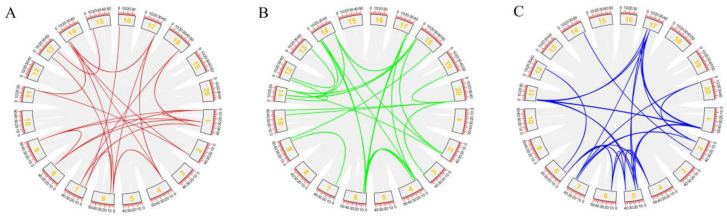
Collinearity analysis of *GmPYLs* (**A**), *GmPP2Cs* (**B**), and *GmSnRK2s* (**C**) gene families. In each circle, red, green, and blue lines showing that most of the *GmPYLs*, *GmPP2Cs*, and *GmSnRK2s* genes existed collinear relationship.

Based on the positioning information for chromosomes in the JGI Phytozome database (Table S1), the result drawing with MapChart software is shown in Figure 4. Among the 20 chromosomes of soybean, 21 *GmPYLs* genes were located on chromosomes 1–4, 6–9, and 13–18. There are three *PYLs* genes on chromosome 1, and two *PYLs* genes on chromosomes 6, 7, 13, and 14; further, the rest of the chromosomes containing a single gene of the *PYL* family. The 36 *GmPP2Cs* genes are located on chromosomes 2–4, 6, 7, 9–14, and 17–20. There are five genes of *GmPP2Cs* on chromosome 6, and the remaining genes are located on other chromosomes. 21 *GmSnRK2s* genes are located on chromosomes 1, 2, 4–8, 11, 12, 14, 17, and 20. The chromosomes 1, 2, 7, 8, 11, and 17 contain two genes, and the rest of the chromosomes contain one gene. Besides, collinearity and replication analysis of all genes was carried out using TBtools software. The results showed that most of the *GmPYLs*, *GmPP2Cs*, and *GmSnRK2s* genes contained a collinear relationship, indicating that most of the *GmPYLs*, *GmPP2Cs*, and *GmSnRK2s* genes had fragment replication, which led to an increase in the number of genes (Figure 5, Table S4). The results showed that *GmPYLs*, *GmPP2Cs*, and *GmSnRK2s* genes had complex genomic replication events, and most of the genes were retained. 

### 2.5. Analysis of GmPYLs, GmPP2Cs, GmSnRK2s Genes Promoter Sequence

**Figure 6:**
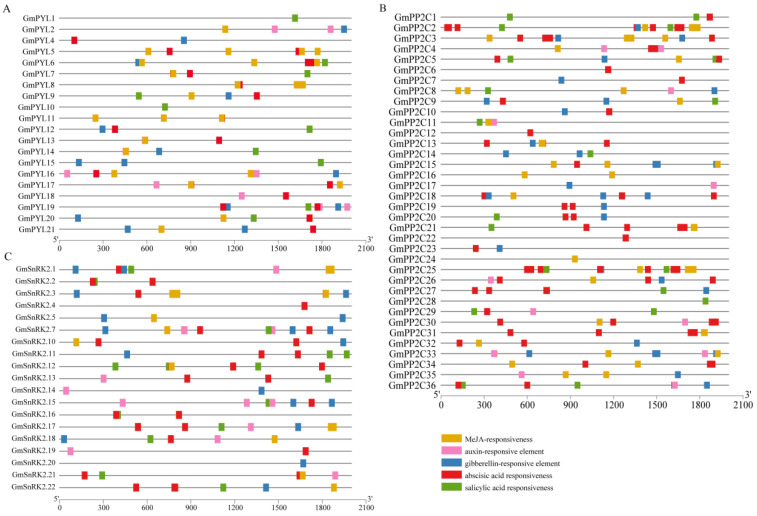
The composition of cis-acting regulatory elements in the promoters of *GmPYLs* (**A**), *GmPP2Cs* (**B**) and *GmSnRK2s* (**C**). The five hormone response elements contained in each gene family promoter include the ABA response element, GA response element, IAA response element, MeJA response element, and the SA response element.

To predict the function of *GmPYLs*, *GmPP2Cs*, and *GmSnRK2s* gene family, the *cis*-acting elements of each *GmPYL-PP2C-SnRK2s* genes’ upstream 2 kb sequences from the transcription start site (TSS) were analyzed. The results show that *GmPYL-PP2C-SnRK2s* gene families have a wide range of potential mechanisms in response to abiotic stress. The promoter region of *GmPYL-PP2C-SnRK2s* gene families mainly contains *cis*-acting elements related to stress, growth, development, and plant hormones. The *GmPYL-PP2C-SnRK2s* gene families contain five hormone response elements. It includes the abscisic acid (ABA) response element, gibberellin response element, auxin response element, MeJA response element, and salicylic acid response element. Between them, mostly, the response elements belong to the abscisic acid (ABA), preceded by the response element of methyl jasmonate (MeJA) (Figure 6, Table S5).

We found eight potential elements to be involved in the regulatory mechanisms of ABA, MeJA, IAA (auxin), GA (gibberellin), and SA (salicylic acid), such as ABRE, TCA-element, AuxRR-core, GARE-motif, CGTCA/TGACG-moif, P-box, TATC-box, and TGA-element. In addition, the four elements (such as ARE, MBS, TC-rich repeats and LTR) were involved in reacting to various stresses, such as drought, low temperature, salinity and anaerobic induction. Various types and numbers of cis-elements have been found in *GmPYLs*, *GmPP2Cs*, *GmSnRK2s* promoters, indicating that they participate in various regulatory mechanisms during plant growth and development (Figure 6, Table S5).

### 2.6. Tissue-Specificity of GmPYL-PP2C-SnRK2s Genes Expression

**Figure 7:**
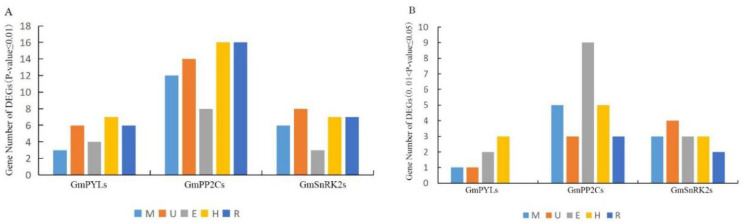
Differentially expressed genes between Williams82 and Jack cultivars in each tissue. Showing the highly significant differential expression (*p*-value ≤ 0.01) (**A**). Showing the significant differential expression (0.01 < *p*-value ≤ 0.05) (**B**). The different color shows different tissue. M, meristem; U, unifoliate leaves; E, epicotyl; H, hypocotyl; R, roots.

**Figure 8:**
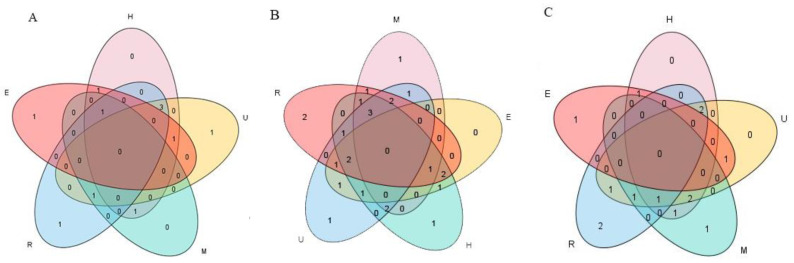
The Venn diagrams represent *GmPYLs* (**A**), *GmPP2Cs* (**B**), and *GmSnRK2s* (**C**), respectively. Showing the number of highly significant differentially expressed genes between Williams82 and Jack, and different circles represent different tissues. The number of overlapping or non-overlapping regions represents the number of genes. M, meristem; U, unifoliate leaves; E, epicotyl; H, hypocotyl; R, roots.

The young seedling of the soybean is more vulnerable to abiotic stress. The two representative soybean cultivars, Williams82 and Jack, were used to extract the total RNA from meristem, unifoliate leaves, epicotyl, hypocotyl, and root at VC stage (After 14 days of seedlings, two unifoliate leaves unroll). Each sample produces 6.0-Gb sequencing data, which is sequenced by a high-throughput transcriptome method to investigate the expression patterns of *GmPYL-PP2C*-*SnRK2s* gene families. The analysis of differentially expressed genes among two varieties showed that the *GmPYLs* gene family contained 2, 6, 4, 7, 6 highly significant differentially genes (*p* ≤ 0.01) and 1, 1, 2, 3, 0 the significant differentially genes (0.01 < *p* ≤ 0.05) in the meristem, unifoliate leaves, epicotyl, hypocotyl, and roots. The *GmPP2Cs* gene family contains 12, 14, 8, 16, 16 highly significant differentially genes and 5, 3, 9, 5, 3 significant differentially genes in the meristem, unifoliate leaves, epicotyl, hypocotyl, and roots, respectively. The *GmSnRK2s* gene family contains 6, 8, 3, 7, 7 highly significant differentially genes, 3, 4, 3, 3, 2 significant differentially genes in the meristem, unifoliate leaves, epicotyl, hypocotyl, and roots respectively (Figure 7). Among them, three genes in the *GmPYLs* gene family, *GmPYL4*, *GmPYL10*, and *GmPYL11*, were significantly different in unifoliate leaves, hypocotyls, and roots. There are three genes in the *GmPP2Cs* gene family, which are *GmPP2C2*, *GmPP2C8*, and *GmPP2C12*, are significantly different in epicotyls, hypocotyls, and roots, the Venn diagram is shown in Figure 8. There is no apparent overlap between the two varieties with significant differences in expression among *GmPYLs*, *GmPP2Cs*, and *GmSnRK2s* gene families. The *p*-value is between 0.01 and 0.05, showing a significant difference between the two groups, while the *p*-value is ≤ 0.01, showing a highly significant difference between the two groups (Table S6).

**Figure 9:**
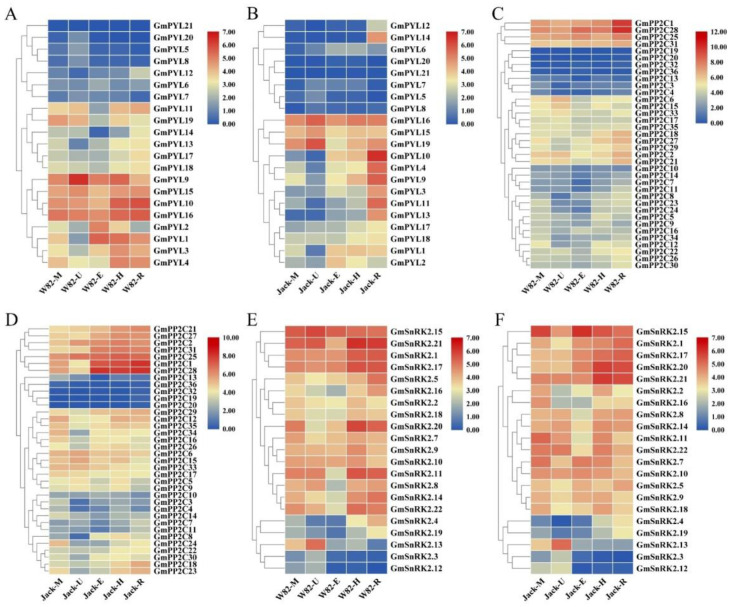
Differentially expressed genes (DEG) in different tissues. *GmPYLs* gene family expression level in Williams82 (**A**) and Jack (**B**). *GmPP2Cs* gene family expression level in Williams82 (**C**) and Jack (**D**). *GmSnRK2s* gene family expression level in Williams82 (**E**) and Jack (**F**). From blue to red represents a gradual increase in value. The left cluster tree is classified according to the level of expression. A horizontal row indicates the expression of the same gene in different tissues. The column represents the expression of different genes in the same tissue.

The tissue-specificity of *GmPYL-PP2C-SnRK2s* gene families’ expression is shown in Figure 9, and the TPM (Transcript Per Million) values are shown in the supplementary file (Table S7). In Williams82 and Jack cultivars, the expression of *GmPYL15* and *GmPYL16* genes were significantly higher in all tissue. Similarly, *GmPP2C1*, *GmPP2C25*, *GmPP2C28*, *GmSnRK2.1*, *GmSnRK15*, *GmSnRK17* and *GmSnRK21* genes were highly expressed in all tissue. It is suggested that these nine genes may play an important regulatory role in response to stress during the early developmental stage.

Among them, three genes in the *GmPYLs* gene family, *GmPYL16*, *GmPYL2*, and *GmPYL14* were significantly different in the unifoliate leaves, hypocotyl, and roots. Five genes of the *GmPP2Cs* gene family were significantly different in the apical meristem, hypocotyl, unifoliate leaves, and in the root, among which the two genes *GmPP2C11* and *GmPP2C29* were significantly different in the root, while *GmPP2C31*, *GmPP2C34*, and *GmPP2C5* had a highly significant difference in the apical meristem, hypocotyl and in unifoliate leaves.

Four genes of the *GmSnRK2s* gene family were significantly different in the meristem, hypocotyl, and root, among which the two genes, *GmSnRK2.5* and *GmSnRK2.16*, significantly different in the root, while the *GmSnRK2.2* and *GmSnRK2.7* are significantly different in meristem and epicotyls.

## 3. Discussion

The *GmPYL-PP2C-SnRK2s* genes are involved in plant abiotic stress responses, which has been documented in many species, as the core genes of the ABA core signaling pathway. In the past 20 years, researchers have revealed how plants react to stress by producing the plant hormone ABA through the physiological and molecular study of the model plant *A. thaliana* under stress conditions. ABA induces various regulatory mechanisms, including gene expression reprogramming, closing stomata, osmotic adjustment, and finally achieving adaptive growth and development [31,32,33]. Studying and implementing the molecular mechanisms on essential cash crops to obtain plant materials with high-stress resistance and sustainable water supply meet global food demand [34]. The ABA signal transduction included three core components of the *GmPYL-PP2C-SnRK2s* gene family [35]. In the presence of ABA, ABA binds with the receptor PYL to form a complex, which binds to PP2C and inhibits the catalytic activity of PP2C by inserting the door ring into the active crack of PP2C. PYL-mediated inhibition of PP2C activates SnRK2 kinases by activating cycloserine autophosphorylation, which transmits ABA signals by phosphorylating downstream transcription factors [8]. The phytohormone ABA plays an important role in plant growth and development, leaf senescence, and lateral root growth [36,37]. In this study, soybean genome and transcriptome data were used to comprehensively analyze the *GmPYLs*, *GmPP2Cs*, and *GmSnRK2s* gene families, which provides a theoretical basis for studying the mechanism of soybean ABA pathway core genes responding to abiotic stress and affecting growth and development [38,39]. Consistent with *A. thaliana* and *O. sativa* [40], the *GmPP2Cs* gene family is larger than *GmPYLs* and *GmSnRK2s*.

The *PYL-PP2C-SnRK2s* gene families support the evolutionary relationship among the three selected species. Among them, the gene location of the *GmSnRK2s* is the same as that of previous studies [26], but compared to other *GmSnRK2s*, *GmSnRK2.6* lacks one intron, three motifs, and some amino acid residues. In addition, *GmSnRK2.6* expression showed no difference at each time point. We analyzed the domains of these 22 *GmSnRK2* genes and found that all of them contained S_TKc domains except *GmSnRK2.6*, the *GmSnRK2.6* contains the Pfam: pkinase domain. Considering these conditions, *GmSnRK2.6* is not included in the analysis of our article. Through phylogenetic analysis, the genes of *GmPYLs*, *GmPP2Cs*, and *GmSnRK2s* families were divided into 3, 10, and 3 groups. In the phylogenetic trees of *G. max*, *A. thaliana* and *O. sativa*, the conserved motifs in the same branch are highly similar, indicating that the same group of genes may have similar functions. For example, multiple genes in *A. thaliana* (*AtPYR1*, *AtPYL1*, *AtPYL2*, *AtPYL3*) all promote ABA-induced stomatal closure [41,42,43].

*SnRK2.6* might influence the expression of RD22 and RD29B, which are ABA-responsive genes in *A. thaliana* [44]. *SnRK2.2*, *SnRK2.6*, and *SnRK2.3* are redundant *SnRK2s* activated by ABA and play important roles in seed development and dormancy regulation [45]. Different studies have shown that PP2C plays an important role in abiotic stress response in plants, especially in the ABA signaling pathway [46,47]. The collinear analysis showed that *GmPYL1*, *GmPYL2*, *GmPYL3*, and *GmPYL4* genes were homologous to the *A. thaliana* ABA-induced stomatal closure genes, it implies the possible similarity in function. All *GmPYLs* genes contain Pfam: Bet_v_1, Pfam:Polyketide_cyc and Pfam: Polyketide_cyc2 domains. Among them, Pfam: Polyketide_cyc and Pfam: Polyketide_cyc2 are members of the START domain superfamily, and their protein folding has a conservative ligand binding mode to adapt to a variety of catalytic activity and non-catalytic regulatory functions. Pfam: Bet_v_1 is the birch pollen protein domain [12], homologous with Pfam: Polyketide_cyc domain [48], overlap with each other in their position. These domains are important for PYL used as the direct receptor of ABA, which combines ABA and PP2C and negatively regulates ABA’s core signal pathway [49]. As a core component of ABA signaling, all *GmPP2Cs* genes contain the PP2Cc domain. In soybean, *GmSnRK2s* genes contain the S_TK domain, which performs an important function in responses to hyperosmotic stress, high salinity, and dehydration [50,51].

The *cis*-acting element analysis results showed that in group III, *GmPYL17*, *GmPYL18*, and *GmPYL19* genes were located at the same branch of the phylogenetic tree, and all contained auxin response elements. Previous studies have shown that *AtPYL8* promotes the transcription of auxin response genes independent of the ABA core signal pathway, relieves the ABA core signal pathway’s inhibition on lateral root growth, and promotes lateral root growth [43]. Phylogenetic analysis showed that *GmPYL17*, *GmPYL18*, and *GmPYL19* genes were firmly related to the *A. thaliana AtPYL8* gene. It is speculated that *GmPYL17*, *GmPYL18*, and *GmPYL19* genes may regulate similar signal pathways in soybean. Besides, all *GmPYLs*, *GmPP2Cs*, and *GmSnRK2s* genes contain at least one plant hormone response element, and plant hormone signals are the key to regulating drought or water deficiency responses [52]. Therefore, *GmPYL-PP2C-SnRK2s* gene families may play an important role in regulating drought or water deficiency responses [53].

Furthermore, previous studies showed that ABA might induce the expression of jasmonate (JA) biosynthetic genes and then increase the concentrations of JA partially based on the function of ABI5 with clade A protein phosphatases type 2C (PP2Cs), consequently leading to the degradation of JAZ proteins [54]. According to Mustilli et al. [55], 10 members of the SnRK2 family in the *A. thaliana* genome, of which ABA strongly induces SnRK2.2, SnRK2.3, and SnRK2.6/OST1. SnRK2.6/OST1 is expressed in stomatal guard cells related to stomatal closure induced by ABA, CO2, and methyl jasmonate (MeJA) [55]. In this study, more than half of the *GmPP2Cs* and *GmSnRK2s* genes contain MeJA response elements, indicating that these genes may be related to stomatal closure.

The differences in expression patterns of *GmPYL-PP2C-SnRK2s* gene families in different soybean tissues indicated that there were functional differences among *GmPYL-PP2C-SnRK2s* gene families. As key genes in ABA’s core signal pathway, *GmPYLs*, *GmPP2Cs*, and *GmSnRK2s* are involved in various abiotic stress responses. It is reported that overexpression of *AtPYR1*, *AtPYL1*, *AtPYL2*, and *AtPYL3* genes in the *A. thaliana* group I can improve drought stress tolerance [13,56]. It can be inferred that group I *GmPYL1*, *GmPYL2*, *GmPYL3*, *GmPYL4*, *GmPYL5*, *GmPYL6*, *GmPYL7*, and *GmPYL8* genes have similar functions, which may also be related to enhance the drought stress tolerance in soybean. The transcriptome data and the previous research data [57] showed the same gene expression patterns, which proves the reliability of the tested results. In the present study, the family structure, evolutionary relationship, and expression pattern of *GmPYLs*, *GmPP2Cs*, and *GmSnRK2s* genes were comprehensively analyzed, which provided a theoretical basis for the directional study of *PYL-PP2C-SnRK2s* genes in soybean.

## 4. Materials and Methods

### 4.1. Plant Materials

Two soybean cultivar, Williams82, and Jack were used as a plant material grown in a greenhouse with (26 °C, light 14 h/dark 10 h) growth conditions. After 14 days of seedlings (VC stage, two unifoliate leaves unroll), the healthy plant’s meristem, unifoliate leaves, epicotyl, hypocotyl, and root were collected separately, and three independent replicas for each sample were frozen into liquid nitrogen. The total RNA was extracted from each tissue using TRIzol (TIANGEN Biotech, Beijing, China) following the manufacturers’ procedure. The total RNA’s quantity/quality was measured with a Nanophotomoter Spectrophotometer (Implen, München, Germany). The whole genomes and amino acid sequences of *A. thaliana*, *G. max*, and *O. sativa* were obtained from JGI Phytozome [58] and Ensembl databases (http://plants.ensembl.org/index.html).

### 4.2. Identification of GmPYL-PP2C-SnRK2s Gene Families

The *AtPYL-PP2C-SnRK2s* gene family information were obtained by using the TAIR database (https://www.arabidopsis.org/) for *A. thaliana.* The JGI Phytozome website (https://phytozome.jgi.doe.gov/pz/portal.html) was used to compare the homology of amino acids, the candidate soybean PYL-PP2C-SnRK2 proteins with high homologous correlation with *A. thaliana* PYL-PP2C-SnRK2 protein. Furthermore, through the NCBI database’s Blastp function, the *GmPYL-PP2C-SnRK2s* genes were identified and given specific names.

### 4.3. Phylogenetic Analysis

The amino acid sequences of GmPYLs, GmPP2Cs, and GmSnRK2s proteins from *G. max*, *A. thaliana*, and *O. sativa* were selected to construct a Maximum Likelihood using MEGA 10.0 software, use “find best DNA / protein models (ML)” program to test the most suitable model. “Jones-Taylor-Thornton (JTT) model” and “Gamma Distributed (G)” rates among sites were selected. The robustness of each node in the tree was determined using 1000 bootstrap replicates, and then the default parameter for the remaining parameters was selected [59].

### 4.4. Analysis of Gene Structure and Their Conservation

The gene structure of *GmPYLs*, *GmPP2Cs*, and *GmSnRK2s* was analyzed online by the GENE Structure display (GSDS 2.0) website (http://gsds.gao-lab.org/). Upload genomic sequence and coding sequence with FASTA format, and then generate gene structure. MEME website (http://meme-suite.org/) and TBtools software (version 1.055) were used to analyze the conserved motif of soybean GmPYLs, GmPP2Cs, and GmSnRK2s protein. The MEME website uses Classic mode to perform motif discovery on protein datasets: site distribution select Zero or One Occurrence Per Sequence (zoops). Generated conserved motif files are visualized with TBtools [60]. Using the plug-in “Batch SMART” of TBtools, it links to the SMART website (http://smart.embl-heidelberg.de/), adopted to determine whether the PYL-PP2C-SnRK2s domain existed in the candidate *PYL-PP2C-SnRK2s* genes [61].

### 4.5. Chromosomal Location and Collinearity Analysis

The chromosome mapping information of *GmPYLs*, *GmPP2Cs*, and *GmSnRK2s* gene families was obtained from the NCBI database. The chromosome mapping map was drawn by MapChart software [62], and the collinear relationship of *GmPYL-PP2C-SnRK2s* gene families was analyzed by using MCScanx and TBtools software. To further elaborate the replication events of the *GmPYL-PP2C-SnRK2s* gene families, and identify orthologous and paralogous genes. 

### 4.6. Analysis of Promoter Sequence

The 2000 bp genomic sequences upstream of the transcription start site (TSS) of *GmPYL-PP2C-SnRK2s* gene families were extracted from the NCBI database, and the *cis*-acting elements in the promoter region were analyzed online by PlantCARE website (http://bioinformatics.psb.ugent.be/webtools/plantcare/html/) [63].

### 4.7. Analysis of Expression Patterns

The expression data were obtained by high-throughput transcriptome sequencing, and the sequencing platform was Illumina Hiseq2500 at Shanghai Biozeron Biological Technology Co., Ltd. Each sample produces 6.0-Gb sequencing data, which is sequenced by double-terminal (PE) sequencing with a reading length of 150 bp. Post-trimming reads were aligned to *G. max* reference genome (Phytozome v11.0, https://phytozome.jgi.doe.gov/pz/portal.html, accessed on 16 July 2016). The transcriptome dataset of soybean (accession number: SRP038111) was uploaded to the National Center for Biotechnology Information (NCBI) Sequence Read Archive (SRA) database. The data used in the chart are the TPM values commonly used in transcriptome analysis, which will be used to analyze the tissue specificity and in different cultivars expression of *GmPYL-PP2C-SnRK2s* gene families. The mapping of these RNA-seq reads was done using TBtools (version 1.055) with the default settings. Quantification of gene expression was done using the TPM algorithm. The clustered heatmap of resulting TPM values per tissue for *GmPYL-PP2C-SnRK2s* was generated using the heatmap function of TBtools (version 1.055). Logarithm TPM data, set base = 2 and log with = 1, and cluster rows.

## 5. Conclusions

It is therefore concluded that there are 21 *PYLs* genes, 36 *PP2Cs* genes, and 21 *SnRK2s* genes present in the entire genome of the soybean. Gene structure, conserved sequence, and domain analysis have shown that members of the *GmPYL-PP2C-SnRK2* gene families are evolutionarily relatively conservative. Complex genomic replication events have occurred; most genes have been retained. Identified *GmPYL-PP2C-SnRK2s* gene families contain START-like domain, PP2Cc domain, and S_TK domain. The expression pattern of these genes has tissue specificity and differences among varieties. In summary, these findings indicate the potential role of *GmPYL-PP2C-SnRK2* gene families in plant growth and development, and multiple stress responses.

## Supplementary Materials

The supplementary materials are available online at https://www.mdpi.com/2223-7747/9/10/1356/s1, Figure S1: The Venn diagrams showing the number of significant differentially expressed genes between Williams82 and Jack. Table S1 *GmPYL-PP2C-SnRK2s* Genes screening, naming and location, Table S2. The accession numbers and gene names of *PYL-PP2C-SnRK2* gene families in *A. thaliana* and *O. sativa*, Table S3. Width, Sites, E-value of *GmPYL-PP2C-SnRK2s* conserved motif, Table S4. The genes that contain a collinear relationship and the location of *GmPYL-PP2C-SnRK2s* on scaffolds, Table S5. Prediction of *cis*-elements of *GmPYL-PP2C-SnRK2s* gene family. Table S6. The P-value of T-test of *GmPYL-PP2C-SnRK2s* gene families DEG Between Williams82 and Jack. Table S7. TPM values of *GmPYL-PP2C-SnRK2s* all genes in two varieties.
