# Supplementary material for: Identification, Evolutionary and Expression Analysis of PYL-PP2C-SnRK2s Gene Families in Soybean
Source: Plants (Basel). 2020 Oct 14;9(10):1356. doi: 10.3390/plants9101356 (PMC7602157; doi:10.3390/plants9101356)
Supplement: Supplementary file 1 [file plants-09-01356-s001.zip › Supplementary data.docx]

**Supplementary data**


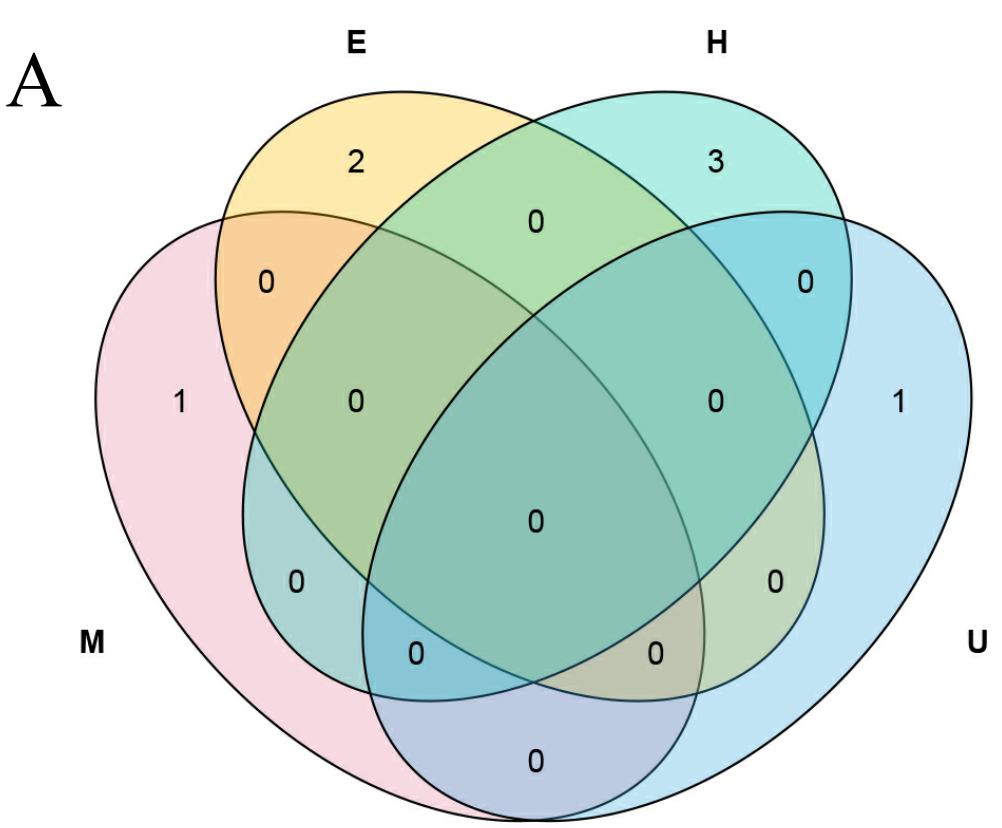

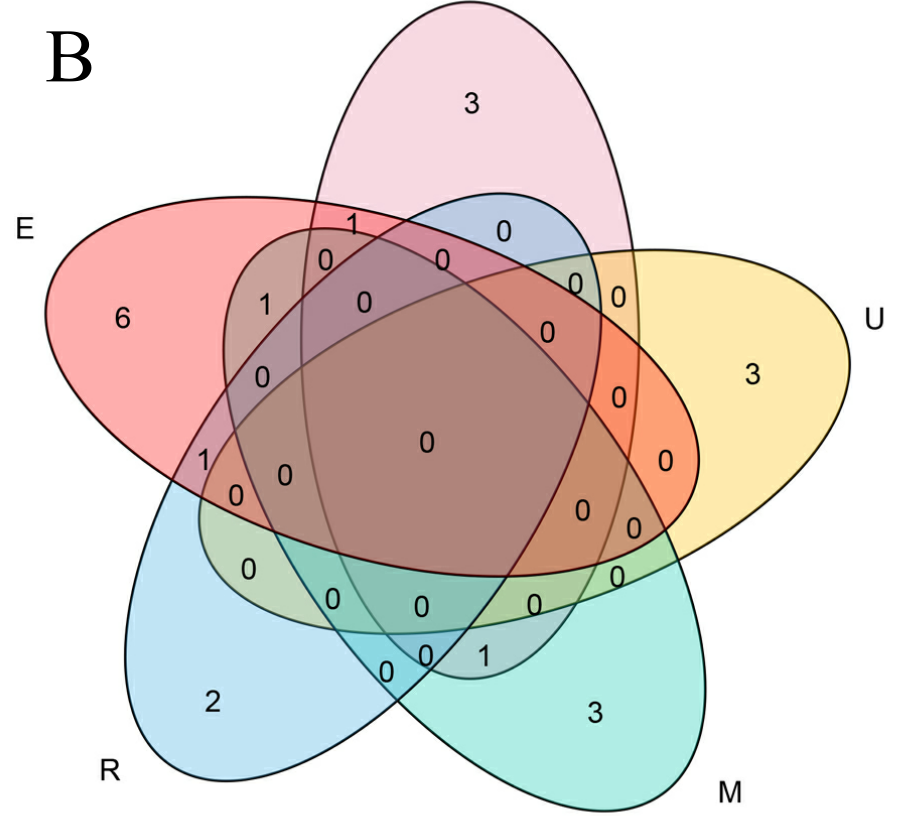

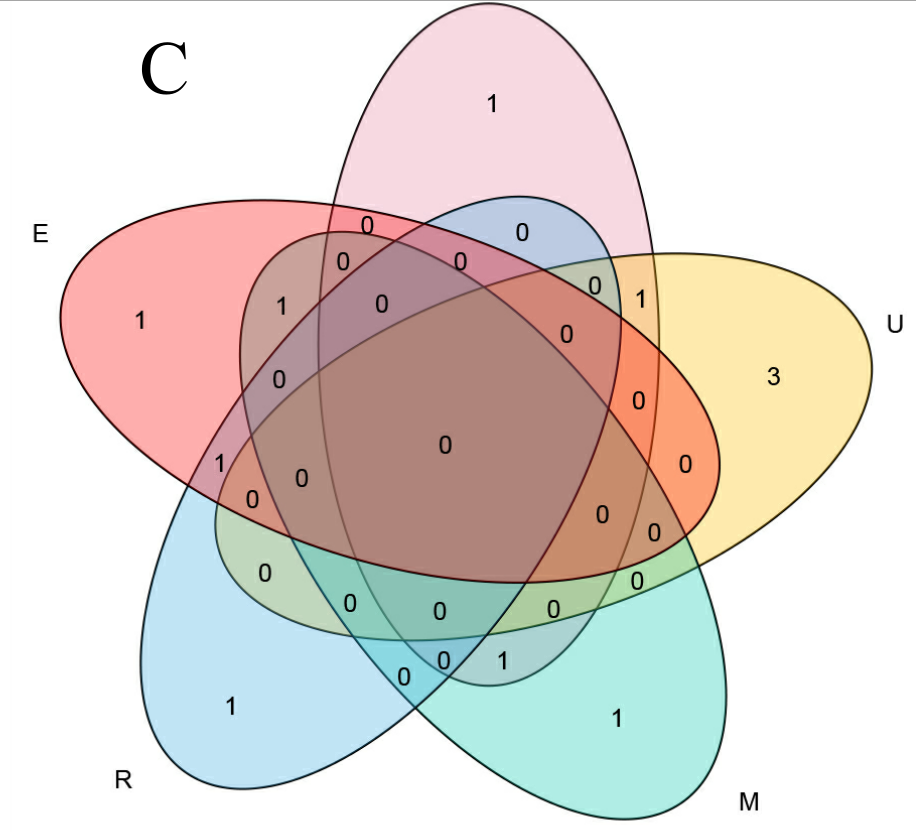


Figure S1 The Venn diagrams represent *GmPYLs* (A), *GmPP2Cs* (B), and *GmSnRK2s* (C), respectively. Showing the number of significant differentially expressed genes between Williams82 and Jack, and different circles represent different tissues. The number of overlapping or non-overlapping regions represents the number of genes. M, meristem; U, unifoliate leaves; E, epicotyl; H, hypocotyl; R, roots.

**Table S1 GmPYLs, GmPP2Cs, GmSnRK2s Genes screening, naming and location**

**Table S2. The accession numbers and gene names of *PYL-PP2C-SnRK2* gene families in Arabidopsis and rice.**

**Table S3. Width,Sites,E-value of GmPYLs, GmPP2Cs and GmSnRK2s conserved motif.**

**Table S4. The genes that contain a collinear relationship and the location of soybean *PP2C-PYL-SnRK2s* on scaffolds.**

**Table S5. Prediction of cis-elements of *GmPYLs-GmPP2C-GmSnRK2* gene family.**

**Table S6. The P-value of T-test of *GmPYL-GmPP2C-GmSnRK2* gene families DEG Between Williams82 and Jack.**

**Table S7. TPM values of *GmPYL-GmPP2C-GmSnRK2* all genes in two varieties.**
